# NF-kappa B mediated Up-regulation of CCCTC-binding factor in pediatric acute lymphoblastic leukemia

**DOI:** 10.1186/1476-4598-13-5

**Published:** 2014-01-07

**Authors:** Han Zhang, Lin Zhu, Huacheng He, Shanshan Zhu, Wei Zhang, Xiao Liu, Xiaoxi Zhao, Chao Gao, Mei Mei, Shilai Bao, Huyong Zheng

**Affiliations:** 1 Beijing Key Laboratory of Pediatric Hematology Oncology; National Key Discipline of Pediatrics, Ministry of Education; Key Laboratory of Major Diseases in Children, Ministry of Education; Hematology Oncology Center, Beijing Children’s Hospital, Capital Medical University, 56 Nanlishi Road, Beijing, 100045, China; 2 State Key Laboratory of Molecular Developmental Biology, Institute of Genetics and Developmental Biology, Chinese Academy of Sciences, West Beichen Road, Beijing 100101, China

**Keywords:** Acute lymphoblastic leukemia, CCCTC binding factor, Cell apoptosis, Cancer

## Abstract

**Background:**

Acute lymphoblastic leukemia (ALL) is the most frequently occurring malignant neoplasm in children. Despite advances in treatment and outcomes for ALL patients, the pathogenesis of the disease remains unclear. Microarray analysis of samples from 100 Chinese children with ALL revealed the up-regulation of *CTCF* (CCCTC binding factor). CTCF is a highly conserved 11-zinc finger protein that is involved in many human cancers; however, the biological function of CTCF in pediatric ALL is unknown.

**Methods:**

The expression patterns of CTCF were evaluated in matched newly diagnosed (ND), complete remission (CR), and relapsed (RE) bone marrow samples from 28 patients. The potential oncogenic mechanism of CTCF and related pathways in leukemogenesis were investigated in leukemia cell lines.

**Results:**

We identified significant up-regulation of CTCF in the ND samples. Importantly, the expression of CTCF returned to normal levels after CR but rebounded in the RE samples. In the pre-B ALL cell line Nalm-6, siRNA-mediated silencing of CTCF expression promoted cell apoptosis and reduced cell proliferation; accordingly, over-expression of a cDNA encoding full-length CTCF protected cells from apoptosis and enhanced cell proliferation. Furthermore, inhibition or activation of the nuclear factor-kappa B (NF-κB) pathway resulted in marked variations in the levels of *CTCF* mRNA and protein in leukemic cells, indicating that CTCF may be involved downstream of the NF-κB pathway. Moreover, inhibition of the NF-κB pathway increased cell apoptosis, which was partially rescued by ectopic over-expression of CTCF, suggesting that CTCF may play a significant role in the anti-apoptotic pathway mediated by NF-κB.

**Conclusions:**

Our results indicate that CTCF serves as both an anti-apoptotic factor and a proliferative factor in leukemic cells. It potentially contributes to leukemogenesis through the NF-κB pathway in pediatric ALL patients.

## Background

Acute lymphoblastic leukemia (ALL) is the most frequent childhood malignancy and accounts for 75% of pediatric leukemias. Since the 1980s, improved supportive care, precise risk classification, and personalized chemotherapy have increased the cure rate of pediatric ALL to approximately 90% in developed countries [1]. However, a better understanding of ALL pathogenesis is needed to further improve the cure rate and quality of life. The advent of high-throughput, genome-wide gene expression analysis has provided new insights into leukemogenesis and suggested potential targets for therapy [2,3].

CCCTC-binding factor (CTCF) is a highly conserved 11-zinc finger protein that is involved in multiple regulatory functions, including transcriptional activation/repression [4,5], chromatin insulation [6,7], DNA imprinting [8,9], and X chromosome inactivation [10,11]. CTCF was first identified and characterized as a transcriptional repressor of the *c-myc* gene in chickens, mice, and humans [5,12,13]. Thus, CTCF was considered as a candidate tumor suppressor. However, CTCF also possesses some oncogenic features. CTCF levels are elevated in breast cancer cell lines and tumors and are associated with resistance to apoptosis [14]. CTCF expression in pediatric leukemia cells has not been investigated.

We previously observed that *CTCF* mRNA levels are up-regulated in leukemic cells based on the genome-wide microarray analysis from 100 Chinese pediatric ALL bone marrow samples [15,16]. To investigate the biological function of CTCF in pediatric ALL, we analyzed CTCF expression in clinical samples at different stages of disease progression and observed CTCF over-expression in leukemic cells from both newly diagnosed (ND) and relapsed (RE) samples. In addition, the expression of CTCF increased in a similar fashion among the different subtypes of pediatric ALL samples and cell lines. Increased CTCF expression in cancer cells could be anti-apoptotic or promote cell proliferation. Using leukemia cell line Nalm-6, we demonstrated that knock-down of CTCF increased cell apoptosis and decreased cell viability; conversely, over-expression of CTCF rescued cells from apoptosis and enhanced cell proliferation. We next explored the mechanistic basis of CTCF function, which revealed that inhibition of nuclear factor-kappa B (NF-κB) activity down-regulated CTCF expression, whereas activation of the NF-κB pathway restored CTCF expression. Furthermore, inhibition of the NF-κB pathway increased cell apoptosis in a process that was partially rescued by ectopic over-expression of CTCF. To this extent, CTCF may contribute to the pathogenesis of pediatric ALL by acting as an anti-apoptotic factor via the NF-κB pathway. These results indicate that CTCF might serve as a possible therapeutic gene target in future clinical strategies.

## Results

### Expression of CTCF in pediatric ALL samples and leukemic cell lines

**Figure 1:**
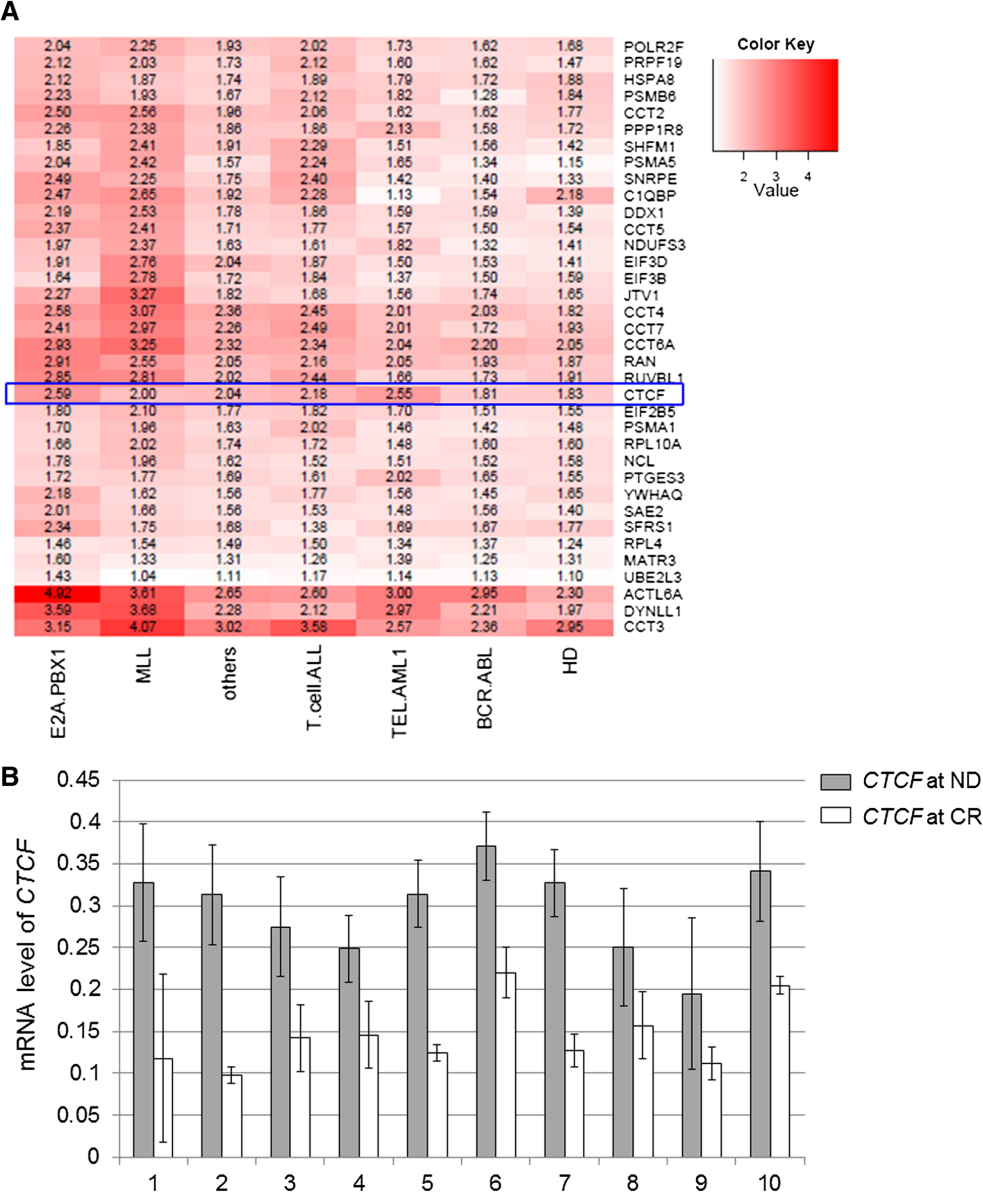
**CTCF levels are dramatically up-regulated in pediatric ALL cases. (A)** A heat map of *CTCF* mRNA levels (blue box). The fold change in expression compared with the control is indicated by the color intensity, with red representing up-regulation. Refer to the additional file 4 of reference [16] for more details. HD, hyperdiploid>50 chromosomes. **(B)***CTCF* mRNA levels were measured by qRT-PCR in paired cDNA samples from 10 ALL patients (n = 20). Each paired sample refers to two samples from the same patient at the time of ND and CR. *CTCF* mRNA levels were increased in the ND samples compared with the CR samples (fold change 2.05, *p* = 0.000, paired samples *t*-test). Each assay was repeated three times. ND, newly diagnosed. CR, complete remission.

Our previous genome-wide microarray analysis of 100 Chinese pediatric ALL cases [15,16] indicated that *CTCF* is up-regulated in leukemia cells (Figure 1A). To validate this finding, we performed qRT-PCR analysis of 10 paired cDNA samples (n = 20) to determine the transcriptional levels of *CTCF*. Each paired sample was obtained from the same patient at the time of new diagnosis (ND) and complete remission (CR). *CTCF* mRNA was elevated in the ND samples compared with the CR samples (Figure 1B and Table 1, fold change 2.05, *p* = 0.000, paired samples *t*-test), consistent with the bioinformatics analysis (Figure 1A).

**Table 1:** Clinical features of the 10 paired pediatric ALL cases and qRT-PCR data

**No.**	**Sex**	**Age of diagnosis (years)**	**Immunotype**	**Cytogenetic abnormality**	**Fusion gene**	**Prognosis**	**mRNA levels of *CTCF***		
							**ND**	**CR**	***p *** **value**
1	M	2	Common B cell	-	-	Remission	0.328	0.118	Paired Samples *T* test: ND-CR, *p* = 0.000
2	M	5	Common B cell	t(12;21)	*TEL-AML1*	Remission	0.313	0.098	
3	M	7	Common B cell	t(1;19)	*E2A-PBX1*	Remission	0.275	0.142	
4	F	3	Common B cell	t(12;21)	*TEL-AML1*	Remission	0.249	0.146	
5	M	2	Common B cell	t(12;21)	*TEL-AML1*	Remission	0.314	0.124	
6	F	3	Common B cell	t(12;21)	*TEL-AML1*	Remission	0.371	0.220	
7	M	4	Common B cell	t(12;21)	*TEL-AML1*	Remission	0.327	0.127	
8	F	3	Common B cell	t(12;21)	*TEL-AML1*	Remission	0.251	0.157	
9	M	3	Pre-B cell	t(12;21)	*TEL-AML1*	Remission	0.195	0.112	
10	F	8	Pro-B cell	-	-	Remission	0.341	0.205	

ND: newly diagnosis; CR: complete remission.

**Figure 2:**
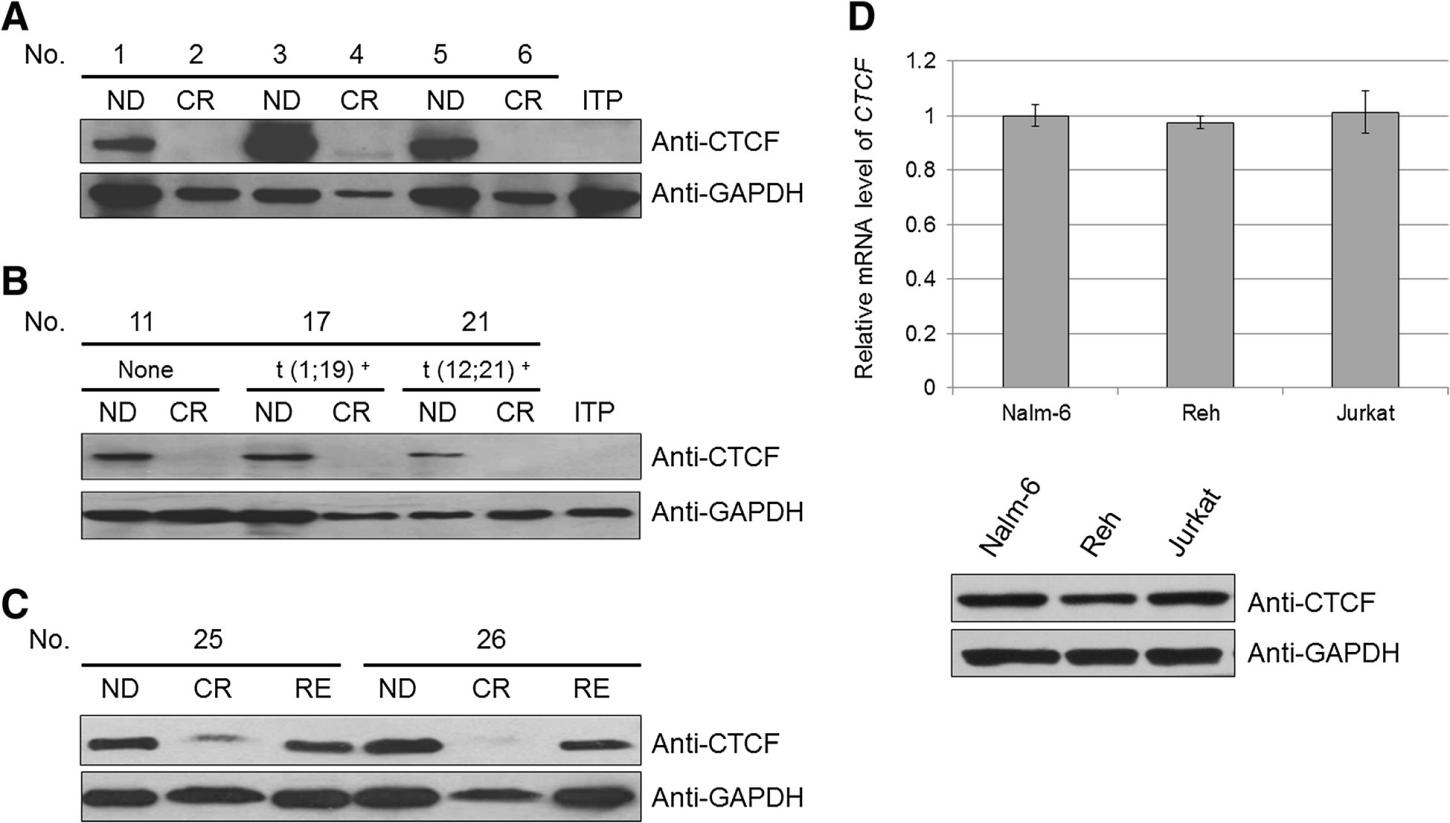
**CTCF expression patterns in pediatric ALL samples and leukemic cell lines. (A)** Western blot analysis of unpaired samples from 8 patients (n = 8, 4 ND and 4 CR). CTCF was over-expressed in the ND samples and displayed reduced expression after CR. A sample from one pediatric patient with immune thrombocytopenic purpura (ITP) was used as a negative control, and GAPDH was used as a loading control. **(B)** Western blot analysis of paired samples taken from 16 pediatric ALL patients (n = 32). The results from 3 patients are shown, including 1 patient with a t(1;19) (*E2A-PBX1*) translocation, 1 with a t(12;21) (*TEL-AML1*) translocation, and 1 without any translocations. CTCF was over-expressed in the ND samples and displayed reduced expression after CR. **(C)** The expression of CTCF rebounded after relapse and maintained normal levels in the CR phase. A total of four relapsed cases were assessed, and the results from two cases are presented. **(D)** The mRNA and protein levels of CTCF in various lymphoblastic leukemia cell types were evaluated by real-time PCR and Western blot. The relative mRNA expression levels were analyzed and presented as mean ± SD from triple replications (*p* = 0.831). Nalm-6 is a pre-B ALL cell line with no fusion gene. Reh is a pre-B ALL cell line with the *TEL-AML1* fusion gene. Jurkat is T lymphocyte cell line. GAPDH was measured as the loading control.

CTCF protein levels were measured by Western blot in samples from 28 patients (n = 52), including 8 unpaired samples (n = 8, 4 ND and 4 CR), 16 ND-CR paired samples (n = 32), and 4 ND-CR-RE matched samples (n = 12) (Table 2). One bone marrow (BM) sample from a patient with immune thrombocytopenic purpura (ITP) was selected as a negative control. CTCF was uniformly expressed at high levels in the ND samples and reduced to normal levels upon CR (Figure 2A, 2B, and 2C). Given the outcome differences among patients with varying cytogenetic abnormalities, we assessed paired samples from different subtypes of ALL, including t(12;21) (*TEL-AML1*), t(1;19) (*E2A-PBX1*), t(9;22) (*BCR-ABL*) (data not shown), and other B-ALL (with no translocation). Similar results were observed among the different subtypes (Figure 2B), indicating that CTCF expression patterns are independent of the cytogenetic subtypes.

**Table 2:** Clinical features of the pediatric ALL cases for bone marrow samples

	**No.**	**Sex**	**Age of diagnosis (years)**	**WBC in PB at newly diagnosis (×10**^**9**^**/L)**	**Immunotype**	**Percentage of blast cells in BM at newly diagnosis (%)**	**Cytogenetic abnormality**	**Fusion gene**	**Prognosis**
Unpaired samples	1	F	10	5.2	Common B cell	89.5	-	-	Remission
	2	M	5	10.5	T cell	98	-	-	Remission
	3	M	2	24	Common B cell	99.5	-	-	Remission
	4	F	7	4.9	Common B cell	99.5	-	-	Remission
	5	M	6	79	Common B cell	93	-	-	Remission
	6	M	6	4.9	Common B cell	96	-	-	Remission
	7	F	10	6.6	Common B cell	96	-	-	Remission
	8	M	5	17.4	Common B cell	90.5	t(12;21)	*TEL-AML1*	Remission
Paired samples	9	F	3	101	Common B cell	99	t(12;21)	*TEL-AML1*	Remission
	10	M	4	84	Common B cell	95	t(12;21)	*TEL-AML1*	Remission
	11	F	8	113	Pro-B cell	97.5	-	-	Remission
	12	M	2	17	Common B cell	89.5	-	-	Remission
	13	M	7	2.4	Common B cell	99	-	-	Remission
	14	F	2	6.8	Common B cell	98	-	*HOX11*	Remission
	15	M	6	18.4	Common B cell	92.5	t(9;22)	*BCR-ABL*	Remission
	16	F	2	6	Common B cell	94.5	t(12;21)	*TEL-AML1*	Remission
	17	M	5	68.2	Common B cell	96	t(1;19)	*E2A-PBX1*	Remission
	18	M	7	6.9	Common B cell	95	t(1;19)	*E2A-PBX1*	Remission
	19	F	8	113	Common B cell	99	t(1;19)	*E2A-PBX1*	Remission
	20	F	3	39.3	Common B cell	96	t(12;21)	*TEL-AML1*	Remission
	21	M	3	65	Pre-B cell	97.5	t(12;21)	*TEL-AML1*	Remission
	22	F	3	14	Common B cell	92.5	t(12;21)	*TEL-AML1*	Remission
	23	M	5	5.4	Common B cell	88.5	t(12;21)	*TEL-AML1*	Remission
	24	M	2	10	Common B cell	87.5	t(12;21)	*TEL-AML1*	Remission
Relapsed samples	25	M	0.58 (7 months)	120	Common B cell	91.5		*MLL*	Dead
	26	M	15	24.5	Common B cell	96	-	-	Dead
	27	M	10	10.3	Common B cell	97.5	-	-	Dead
	28	M	9	14	Common B cell	98	-	-	Dead

WBC, white blood cell; PB, peripheral blood; BM, bone marrow.

To investigate the expression features of CTCF in relapsed patients, samples were collected from 4 relapsed ALL patients. Interestingly, CTCF expression levels increased again after disease relapse (Figure 2C), suggesting that CTCF might be a sensitive biomarker that is predictive of relapse.

In addition to the clinical samples, we further determined the expression features of CTCF in various human lymphoblastic leukemia cell lines. Two B-lineage ALL (B-ALL) cell lines Nalm-6 and Reh, and one T-lineage ALL (T-ALL) cell line Jurkat were evaluated. The Nalm-6 cell line contains no fusion gene, whereas the Reh cell line carries the *TEL-AML1* fusion gene. As shown in Figure 2D, no differences of *CTCF* mRNA levels were observed among three types of leukemia cells (*p* = 0.831, one-way ANOVA). Accordingly, CTCF protein in these cells displayed similar expression patterns, consistent with the change in clinical samples. In view of the fact that approximately 85% of pediatric ALL cases are B-ALL and nearly 80% cases are with no chromosomal and molecular genetic abnormalities [1], Nalm-6 is selected to further explore the potential oncogenic mechanism of CTCF in leukemogenesis.

### The effect of CTCF knock-down on apoptosis and proliferation in leukemic cells

**Figure 3:**
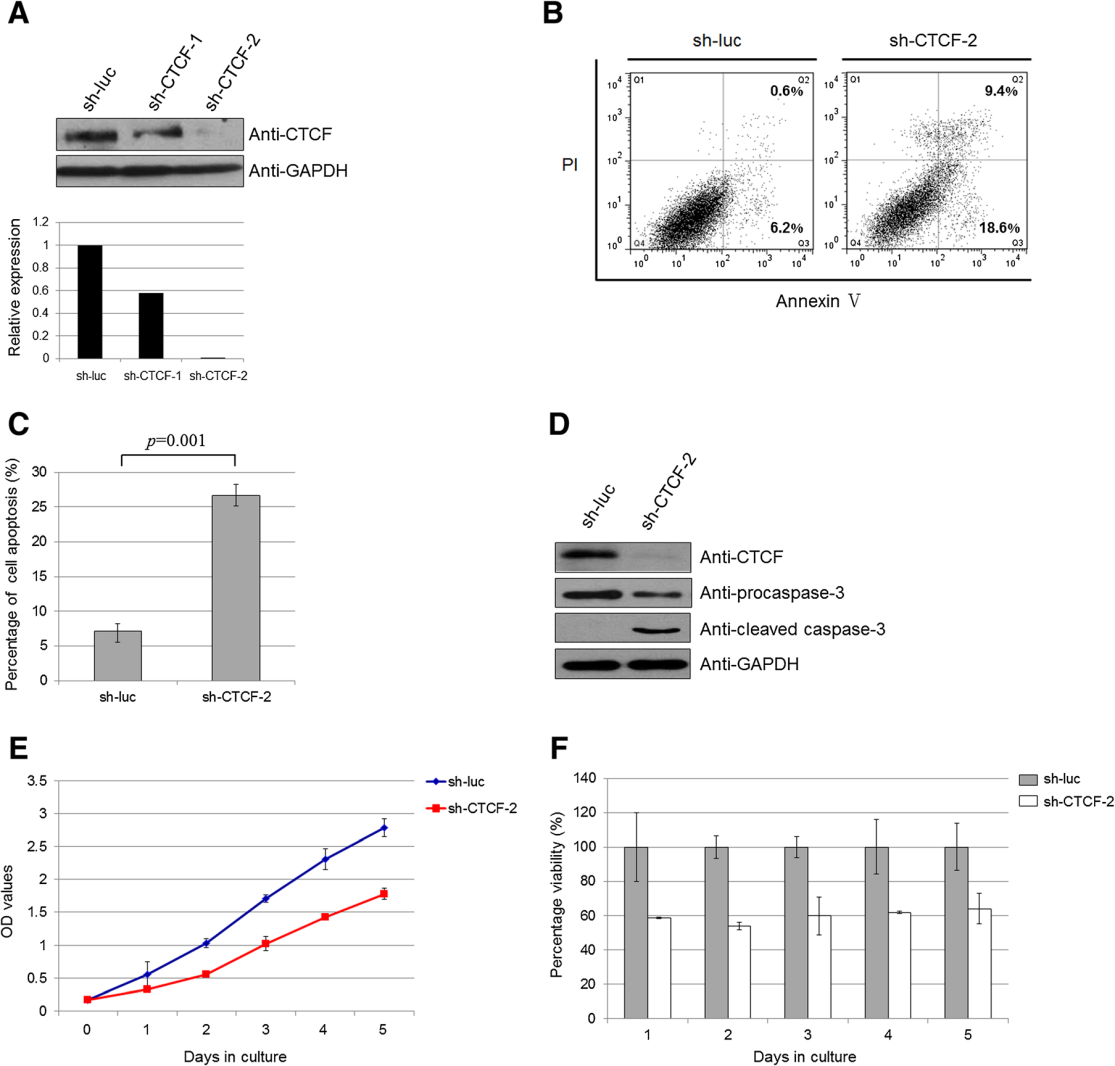
**The effect of CTCF knock-down on apoptosis and proliferation in leukemic cells. (A)** sh-CTCF-1 and sh-CTCF-2 are shRNA plasmids specific for CTCF. sh-luc (targeting firefly luciferase) is the shRNA plasmid specific for firefly luciferase and was used as a negative control. CTCF expression levels were semi-quantified by analysis of the Western blot with Gel-Pro Analyzer software. The sh-CTCF-2 plasmid showed better knock-down efficiency than the sh-CTCF-1 plasmid. **(B)** Knock-down of CTCF resulted in increased early cell apoptosis [Annexin V (+)/PI (-)] and late cell apoptosis [Annexin V (+)/PI (+)]. The percentages of cells in early and late apoptosis are indicated in the figure, respectively. sh-luc was used as a control. **(C)** The percentage of cell apoptosis (both early and late apoptosis) in each group is shown in the diagram as mean ± SD from three independent experiments (*p* = 0.001). **(D)** Effect of knocking down *CTCF* mRNA on caspase-3 activation in Nalm-6 cells. The expression levels of procaspase-3, cleaved caspase-3 and transfected plasmids were determined using Western blot. **(E)** Knock-down clones were seeded in a 96-well plate at 1 × 10^4^ cells per well, with three replicates per clone. Viable cells were counted using the CCK-8 assay for 5 days. **(F)** The percentage of cell viability normalized to nonspecific sh-luc is presented as mean ± SD from three independent experiments.

The high expression of the zinc finger protein CTCF in leukemic cells prompted us to investigate whether CTCF could affect apoptosis or proliferation in lymphoblastic cells. To further determine the effect of altered CTCF activity on cell apoptosis and proliferation, two CTCF shRNA-expressing plasmids (sh-CTCF-1 and sh-CTCF-2) were constructed. The shRNA plasmid specific for firefly luciferase (sh-luc) was used as a control. The RNA interference efficiency of the shRNAs was evaluated by Western blot and parallel semi-quantitative analysis in Nalm-6 cells. Our data revealed that sh-CTCF-2 was more effective at knock-down than sh-CTCF-1 (Figure 3A); hence, sh-CTCF-2 was selected for the subsequent assays.

Cellular apoptosis was detected at 72 h after transfection of the shRNA plasmids. Because all the plasmids carried a GFP tag, we detected cell apoptosis in GFP-positive cells sorted by flow cytometry. As expected, CTCF knock-down by sh-CTCF-2 resulted in a 2-fold increase in early cell apoptosis and an approximately 15-fold increase in late cell apoptosis (Figure 3B and 3C). Consistent with these findings, CTCF knock-down by sh-CTCF-2 decreased the expression of inactive procaspase-3, and triggered the activation of cleaved-caspase-3, indicating that inhibition of CTCF leads to activation of the apoptotic pathway in leukemic cells (Figure 3D).

We next sought to determine whether CTCF knock-down has deleterious effects on cell viability. Leukemic cells treated with CTCF-specific shRNAs consistently showed at least a 40% decrease in cell viability (Figure 3E and 3F, *p* = 0.018, paired samples *t*-test). These data support the notion that CTCF activity is involved in both leukemic cell death and proliferation.

### The effect of CTCF over-expression on apoptosis and proliferation in leukemic cells

**Figure 4:**
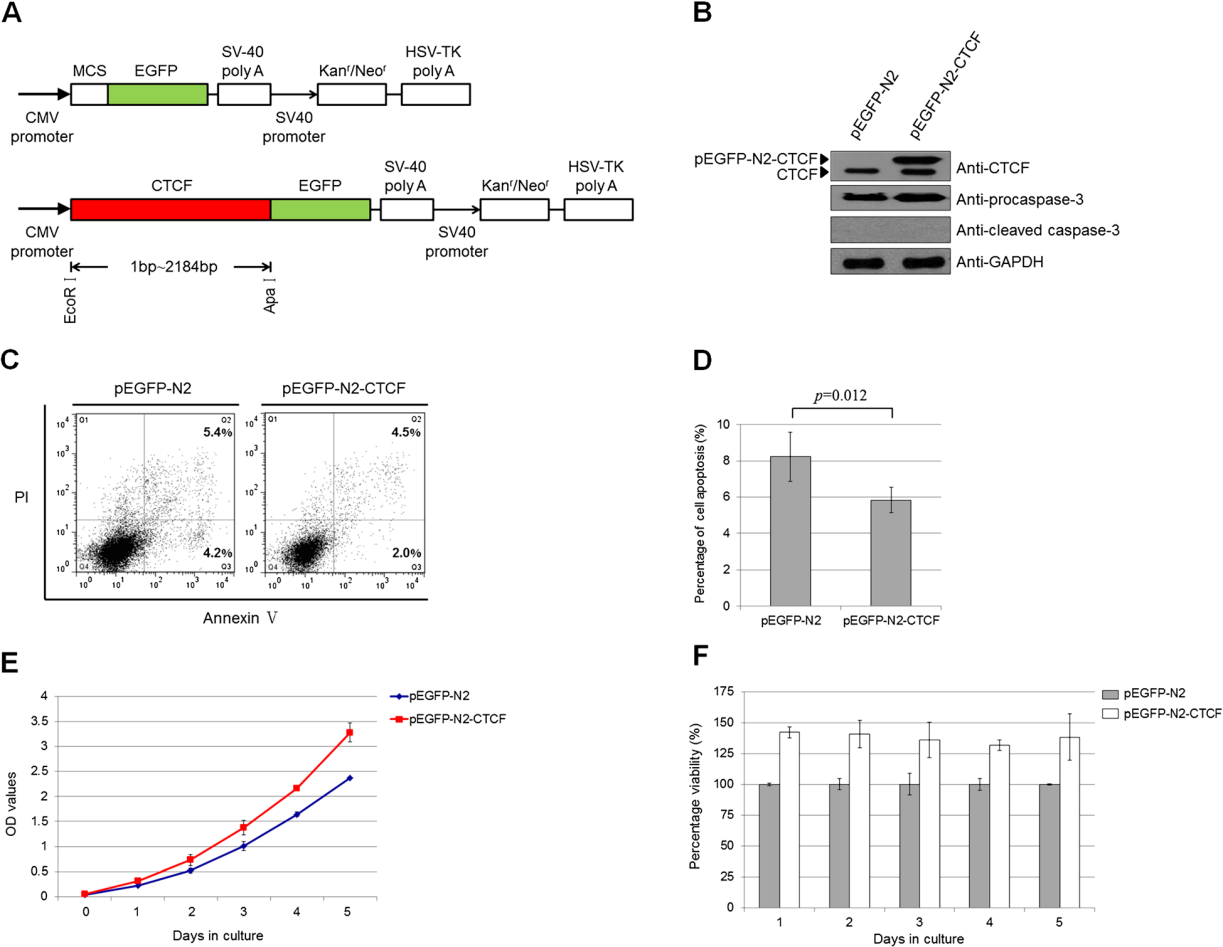
**The effect of CTCF over-expression on apoptosis and proliferation in leukemic cells. (A)** A schematic outline of the pEGFP-N2-CTCF expression vector. The full-length CTCF gene was cloned into the pEGFP-N2 vector. **(B)** Effect of over-expressing CTCF on caspase-3 activation in Nalm-6 cells. The expression levels of procaspase-3, cleaved caspase-3 and the transfected plasmids were assessed by Western blot. **(C)** Over-expression of CTCF resulted in decreased early cell apoptosis [Annexin V (+)/PI (-)] and late cell apoptosis [Annexin V (+)/PI (+)]. The percentages of cells in early and late apoptosis are indicated in the figure, respectively. pEGFP-N2 vector was used as a control. **(D)** The percentage of cell apoptosis (both early and late apoptosis) in each group is indicated in the diagram as mean ± SD from three independent experiments (*p* = 0.012). **(E)** Clones over-expressing CTCF were seeded in a 96-well plate at 1 × 10^4^ cells per well, with 3 replicates per clone. Viable cells were counted using the CCK-8 assay for 5 days. **(F)** The percentage of cell viability normalized to the empty vector pEGFP-N2 is shown in the diagram.

To determine whether CTCF over-expression could rescue leukemic cells from the induction of apoptosis, the over-expression plasmid pEGFP-N2-CTCF was constructed from an in-frame fusion of CTCF and an enhanced-GFP tag (Figure 4A). In this experiment, pEGFP-N2-CTCF and the empty plasmid pEGFP-N2 were transiently transfected into the leukemic cell line. Expression levels from the transfected plasmids (pEGFP-N2-CTCF and pEGFP-N2) were assessed by Western blot (Figure 4B). As shown in Figures 4C and 4D, ectopic over-expression of CTCF rescued approximately 50% of early apoptotic cells from apoptosis. Additionally, the effect of over-expressed CTCF on cell apoptosis was further determined by analyzing caspase-3 activities. However, over-expression of CTCF in transfected Nalm-6 cells resulted in a counter effect against caspase-3 activation, and no cleaved caspase-3 was detected (Figure 4B).

In the subsequent experiment, we assessed the effect of CTCF over-expression on leukemic cell proliferation. An approximately 30% increase in cell viability was observed (Figure 4E and 4F, *p* = 0.047, paired samples *t*-test). However, the changes in cell apoptosis and proliferation were not as prominent as those observed in the knock-down experiments, primarily due to increased basal levels of CTCF in leukemic cells. These results demonstrate that CTCF serves as both an anti-apoptotic and proliferative factor in leukemic cells.

### The effect of NF-κB pathway inhibition on CTCF expression in leukemic cells

**Figure 5:**
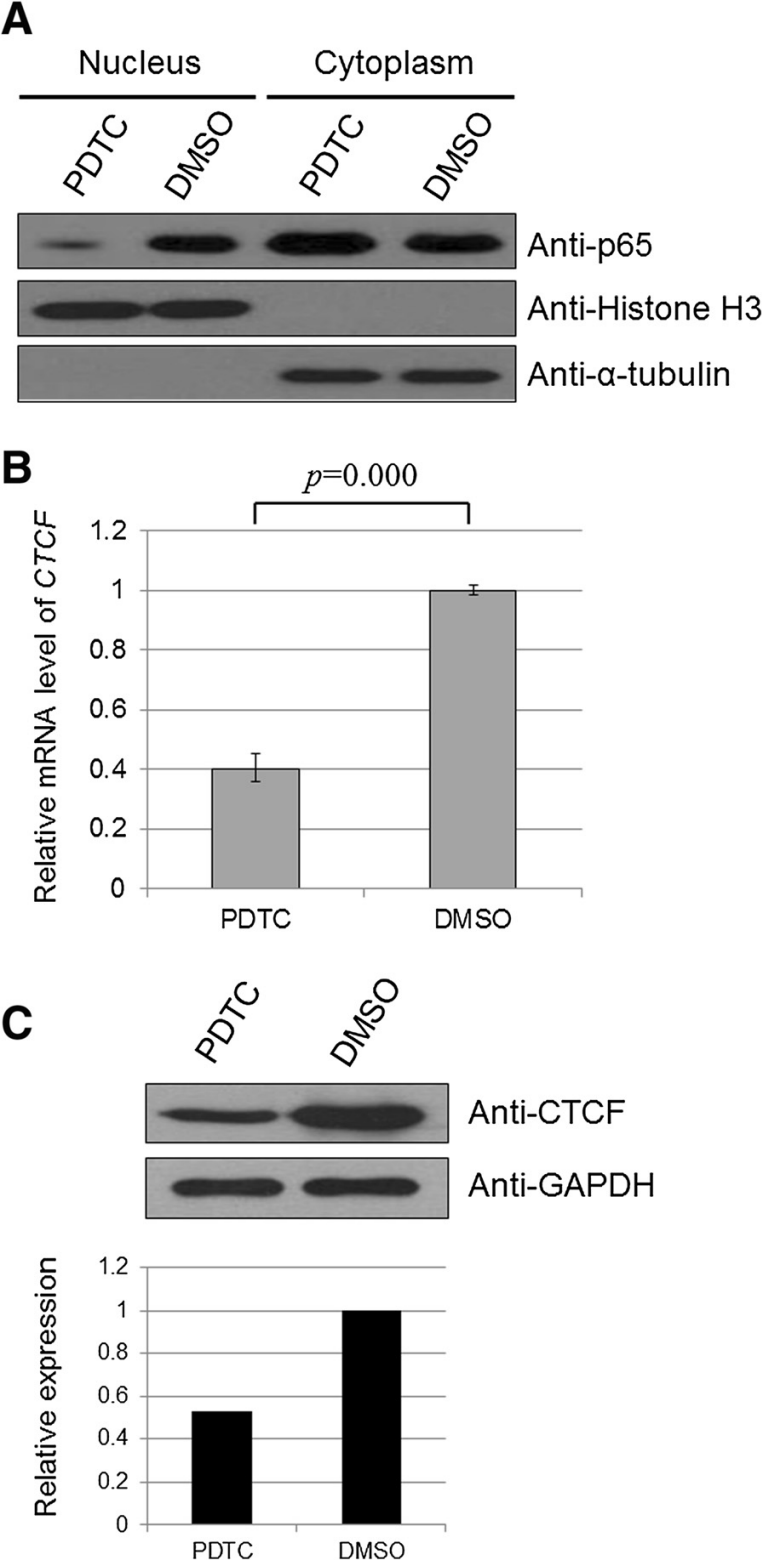
**Expression of CTCF in leukemic cells before and after treatment with an inhibitor of NF-κB activity. (A)** Nalm-6 cells were treated with the NF-κB inhibitor PDTC or DMSO (negative control) for 20 h. NF-κB pathway activity was detected by Western blot using a nuclear p65-specific antibody. Histone H3 and α-tubulin were used as the loading controls for nuclear and cytoplasmic proteins, respectively. **(B)***CTCF* mRNA levels in cell lysates were measured by real-time PCR. The relative changes in the expression levels were analyzed and presented as mean ± SD from triple replications (fold change 2.49, *p* = 0.000). **(C)** Cell lysates were probed with an anti-CTCF antibody, and the expression levels of CTCF were semi-quantified by analyzing the Western blot with Gel-Pro Analyzer software (fold change 1.89). GAPDH was used as a loading control.

The transcription factor NF-κB is involved in many key cellular processes and has emerged as a major regulator of programmed cell death (PCD) via apoptosis or necrosis [17]. Increasing evidence suggests that NF-κB plays an important role in tumorigenesis. NF-κB possesses important regulatory functions for both normal and malignant hematopoiesis and is constitutively activated in pediatric ALL samples [18]. Therefore, we investigated whether the NF-κB pathway is involved in the anti-apoptotic or proliferative effects of CTCF in leukemic cells. To test this hypothesis, the effect of NF-κB on CTCF expression was examined via inhibition of NF-κB activity with ammonium pyrrolidinedithiocarbamate (PDTC), a NF-κB specific inhibitor. The activation of the NF-κB pathway was evaluated by Western blot using a specific antibody against nuclear p65 (Figure 5A). After inhibition of NF-κB activity, *CTCF* mRNA levels decreased (Figure 5B, fold change 2.49, *p* = 0.000). CTCF protein levels were down-regulated accordingly, consistent with the change in mRNA levels (Figure 5C, fold change 1.89). These data indicate that CTCF is likely involved downstream of the NF-κB pathway in leukemic cells.

### The effect of NF-κB pathway activation on CTCF expression in leukemic cells

**Figure 6:**
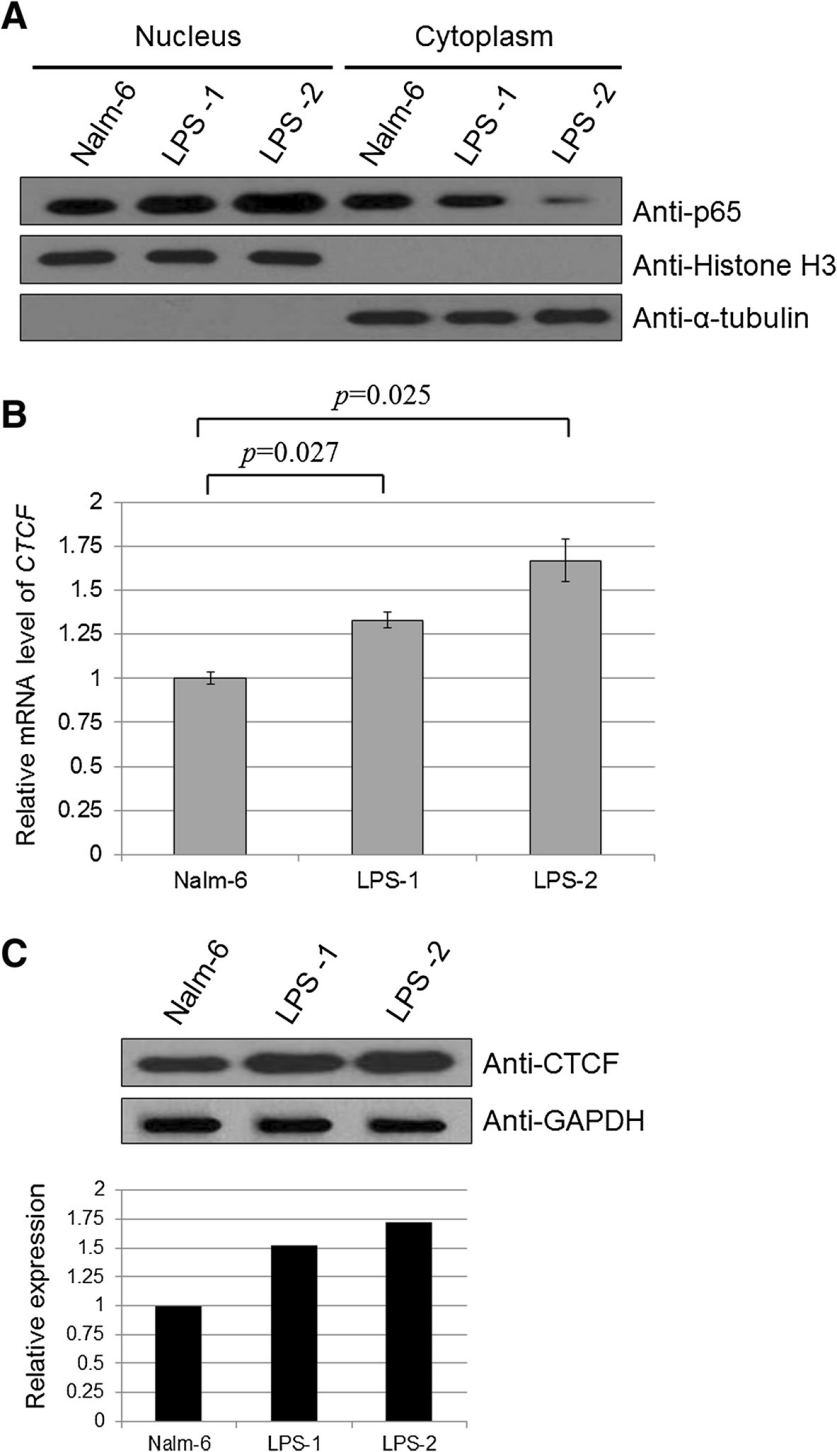
**Expression of CTCF in leukemic cells before and after treatment with an activator of NF-κB activity. (A)** Nalm-6 cells were treated with different doses (5 or 10 μg/ml) of the NF-κB activity activator LPS for 12 h. NF-κB pathway activity was detected by Western blot using a nuclear p65-specific antibody. Histone H3 and α-tubulin were used as the loading controls for nuclear and cytoplasmic proteins, respectively. **(B)***CTCF* mRNA levels in cell lysates were measured by real-time PCR. The relative changes in the expression levels after treatment with 5 and 10 μg/ml LPS were analyzed and presented as mean ± SD from triple replications (fold change 1.33, *p* = 0.027; fold change 1.66, *p* = 0.025, respectively). **(C)** Cell lysates were probed with an anti-CTCF antibody, and the expression levels of CTCF were semi-quantified by analyzing the Western blot with Gel-Pro Analyzer software. The fold changes in expression after treatment with 5 and 10 μg/ml LPS were 1.53 and 1.72, respectively. GAPDH was used as a loading control.

To further elucidate the regulatory role of CTCF in the NF-κB pathway, we treated Nalm-6 cells with different concentrations (5 or 10 μg/ml) of lipopolysaccharide (LPS), a potent activator of the NF-κB pathway [19]. The nuclear translocation of NF-κB p65 was enhanced with increasing LPS concentrations, indicating that the NF-κB pathway was activated effectively by LPS (Figure 6A). As shown in Figure 6B, NF-κB pathway activation with 5 or 10 μg/ml LPS resulted in a 1.33-fold (*p* = 0.027, paired samples *t*-test) and 1.66-fold (*p* = 0.025, paired samples *t*-test) increase in *CTCF* mRNA, respectively. Correspondingly, the protein level increased in a dose-dependent manner by 1.53-fold and 1.72-fold, respectively (Figure 6C). These data demonstrate that CTCF is regulated by NF-κB factors and may play a role downstream of the NF-κB pathway.

### The effect of CTCF on cell apoptosis and proliferation in PDTC-induced leukemic cells

**Figure 7:**
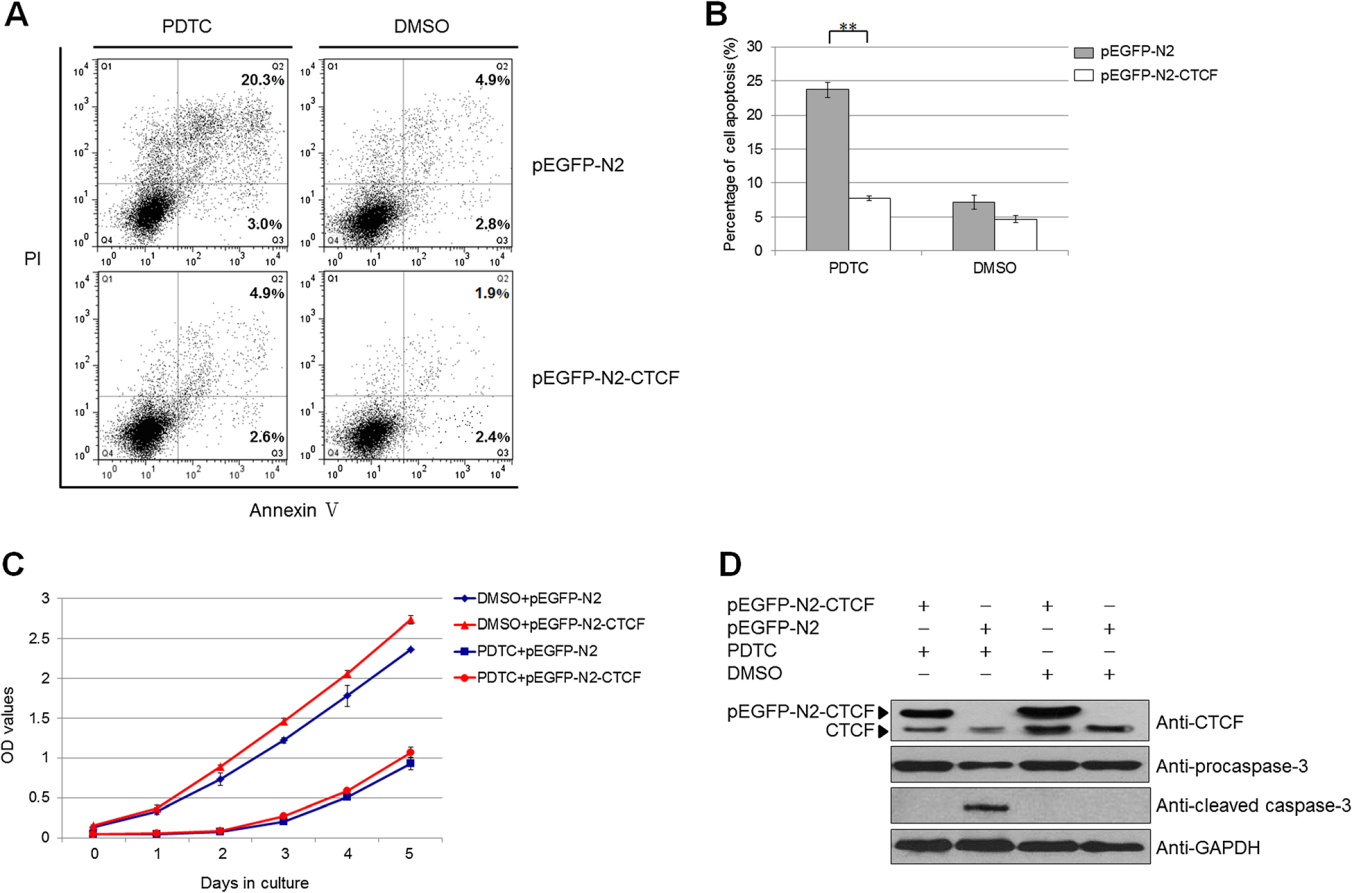
**Over-expression of CTCF rescues the cell apoptosis and proliferation induced by an NF-κB activity inhibitor. (A)** Nalm-6 cells were treated with the NF-κB inhibitor PDTC or DMSO for 20 h before transient transfection with the over-expressing plasmids. Cellular apoptosis was assessed by flow cytometry analysis of Annexin V-APC/PI staining at 48 h after transfection. The percentages of cells in early apoptosis [Annexin V (+)/PI (-)] and late apoptosis [Annexin V (+)/PI (+)] are indicated in the figure, respectively. **(B)** The percentage of cell apoptosis (both early and late apoptosis) in each group is indicated in the diagram as mean ± SD from three independent experiments (**p = 0.000). **(C)** Clones over-expressing CTCF were seeded in a 96-well plate at 1 × 10^4^ cells per well, with three replicates per clone. Viable cells were counted using the CCK-8 assay for 5 days. **(D)** Caspase-3 activation and the expression levels of the transfected plasmids were measured by Western blot.

As previously shown, CTCF knock-down significantly increased Annexin V staining of Nalm-6 cells and decreased cell viability. To further determine whether CTCF was involved in the apoptotic or proliferative pathway mediated by NF-κB, we over-expressed CTCF in combination with treatment with PDTC or DMSO. After a 20-h pre-treatment of Nalm-6 cells with PDTC or DMSO, the over-expressing plasmids were transiently transfected; cellular apoptosis and viability were detected 48 h after transfection. Interestingly, the results demonstrated that ectopically over-expressed CTCF partially rescued the Annexin V stained cells, particularly the late apoptotic cells induced by the NF-κB-inhibitor PDTC (Figure 7A and 7B). By contrast, CTCF over-expression did not rescue the PDTC-induced proliferative inhibition of leukemic cells (Figure 7C, *p* = 0.070, paired samples *t*-test). Caspase-3 assay and the expression levels of the transfected plasmids were also assessed by Western blot (Figure 7D). These data imply that CTCF plays an important role in the anti-apoptotic pathway mediated by NF-κB factors.

## Discussion

CTCF functions as an epigenetic regulator and transcription factor that controls gene expression and cell fate. In B cell lymphomas, increased expression of CTCF is associated with down-regulation of c-myc, resulting in cell growth arrest and apoptosis [20]. Accumulation of CTCF in human K562 myeloid cells leads to growth inhibition and promotion of differentiation into the erythroid lineage [21]. Ectopic expression of CTCF in many cell types inhibits cell clonogenicity by causing growth retardation without apoptosis [22]. Sufficient evidence proves that *CTCF* could be a tumor suppressor gene. However, other studies have provided evidence contradicting a pro-apoptotic or anti-proliferative role of CTCF. CTCF knock-down triggers apoptosis in breast cancer cells, whereas over-expression of CTCF partially protects cells from Bax-induced apoptosis [14]; *CTCF* mRNA knock-down promotes stress-induced apoptosis in human corneal epithelial cells [23]. These contradictory results led us to investigate the biological function of CTCF in pediatric ALL.

Our previous genome-wide microarray analysis of samples from 100 children with ALL revealed that *CTCF* mRNA was over-expressed. The present study revealed that the mRNA and protein levels of CTCF are up-regulated in ND samples and return to normal levels in CR samples following chemotherapy, suggesting that CTCF may serve as a promising indicator of disease progression and treatment response.

Although intensive chemotherapy combined with potent supportive care has improved the survival conditions of pediatric ALL patients, the overall cure rate has not significantly increased in recent years. Approximately 20% of patients relapse, a leading factor in treatment failure. Notably, this study revealed CTCF expression signatures associated with disease relapse. A total of 4 relapsed ALL patients were enrolled in this study to observe the changes in CTCF expression during different disease phases. We observed that CTCF expression increased again upon disease relapse but remained at normal levels in the CR samples. This finding indicates that CTCF levels increased as the malignant clones expanded and were detectable for a brief time before disease recurrence. Additional clinical samples should be studied to confirm these findings.

Leukemia is recognized as a progressive, malignant disease caused by distorted differentiation, apoptosis, and proliferation of hematopoietic cells at different stages. The levels of CTCF were elevated rather than decreased in pediatric ALL samples, which is not characteristic of a tumor suppressor and inspired us to further examine this finding. We hypothesized that over-expression of CTCF may protect leukemic cells from apoptotic cell death or promote cancer cell proliferation. As expected, reduced CTCF levels caused apoptotic cell death and proliferative inhibition in leukemic cell lines. These results indicate a possible link between CTCF expression and sensitivity to apoptosis and proliferation. Specifically, increased CTCF levels may be necessary to protect against apoptotic stimuli and promote leukemic cell viability. These findings may be relevant to the potential use of CTCF as a therapeutic target in pediatric ALL because reducing CTCF levels could result in apoptotic cell death and growth inhibition of cancer cells without affecting normal blood cells, although further studies are needed.

CTCF over-expression has been reported to induce apoptosis and growth retardation in various cell types [20-22]. Undoubtedly, our results introduce a controversial role for the tumor suppressor CTCF in apoptosis and proliferation. Cell type, cellular environment, genetic background, and other variables play important roles in the ultimate function of CTCF. The combination of these factors often has conflicting effects, making it difficult to predict the exact functional outcome of any combination. For example, WT-1 [24-27] may behave as either an anti-apoptotic or pro-apoptotic factor in different cellular contexts. Previous study reported such a controversial role of CTCF in breast cancer cells [14] and human corneal epithelial cells [23], which strongly support our findings. In this study, we suggest a similar dual role for CTCF in pre-B ALL cells.

Explanation of this complex behavior will require a better understanding of regulatory networks. Increased CTCF levels in leukemic cells may be involved in the development of apoptotic resistance and increased cell proliferation. NF-κB is a multi-component pathway that controls hundreds of genes involved in diverse cellular processes, including cell proliferation, cellular growth, and apoptosis. Dysregulation of the pathway leads to many human diseases, such as cancer [28]. Here, we demonstrated that changes in NF-κB activation by either a NF-κB-inhibitor or a NF-κB-activator affected *CTCF* mRNA and protein expression in leukemic cells, suggesting that CTCF is involved downstream of the NF-κB pathway.

To further explore the functional role of CTCF in the NF-κB pathway, we determined that ectopic over-expression of CTCF effectively rescues Nalm-6 cells from apoptotic death rather than proliferative inhibition, indicating that CTCF is primarily involved in the anti-apoptotic pathway mediated by NF-κB in leukemic cells. However, the ability of CTCF to rescue cells from apoptotic death induced by an NF-κB-inhibitor cannot be explained solely by this pathway. The regulation of cell apoptosis and proliferation by CTCF also involves other pathways, such as the extracellular signal-regulated kinase (Erk) and Akt signaling pathways [29]. Further studies are needed to clarify the direct and indirect effects of CTCF on this regulation. In addition, several unanswered questions must be addressed, including which NF-κB subtype interacts with CTCF in leukemic cells and which network regulates CTCF involvement in the NF-κB signaling pathway.

## Conclusions

In this paper, which is the first to link the zinc finger protein CTCF with pediatric ALL, the following conclusions are made. 1) CTCF expression patterns could serve as a sensitive indicator of CR and RE in ALL. 2) Our results support the hypothesis that increased levels of the CTCF protein can protect leukemic cells against apoptosis and promote cell proliferation, indicating that CTCF is a promising target for anti-leukemic therapy. 3) CTCF is involved downstream of the NF-κB signaling pathway and plays an important role in the anti-apoptotic pathway mediated by NF-κB. In future studies, additional samples and regulatory network research will be investigated to elucidate the role of CTCF in pediatric ALL.

## Materials and methods

### Patient information

A total of 28 children (7 months to 15 years, median age of 5 years) diagnosed with ALL and treated in the Hematology Oncology Center of Beijing Children’s Hospital between December 2002 and April 2009 were enrolled in this study. Informed consent was obtained from the parents or legal guardians of the patients. A single sample was obtained from a child with immune thrombocytopenic purpura (ITP) as a negative control. The study design followed the Helsinki guidelines and was approved by the Beijing Children’s Hospital Ethics Committee prior to initiating the study.

All patients were diagnosed with ALL using a combination of morphology, immunology, cytogenetics, and molecular biology (MICM). The cytogenetic ALL subtypes were experimentally identified by G-banding karyotype and multiplex nested reverse transcription-polymerase chain reaction (RT-PCR). A total of 29 fusion genes were assessed by RT-PCR, including *TEL-AML1*, *BCR-ABL*, *E2A-PBX1*, *MLL-AF4*, and *SIL-TAL1*.

Paired bone marrow (BM) samples from 16 pediatric patients (n = 32) were collected at the time the patient was characterized as newly diagnosed (ND) or in complete remission (CR). From these samples, 10 (n = 20) were randomly selected for quantitative real-time PCR (qRT-PCR) analysis (Table 1). In addition, 8 unpaired BM samples (n = 8) from 4 ND and 4 CR patients were collected. Matched BM samples were also collected from 4 relapsed patients at the time of ND, CR, and relapse (RE) (n = 12). The clinical features of these patients are described in detail in Table 2.

### Cell samples, RNA isolation, and qRT-PCR

BM samples were collected in ethylenediaminetetraacetic acid (EDTA) tubes. Mononuclear cells were isolated by Ficoll gradient centrifugation (MD Pacific, Tianjin, China, density: 1.077 g/ml) and cryo-preserved in a -80°C freezer for subsequent experiments. Total RNA from the BM samples and cell lines was extracted using Trizol reagent (Invitrogen, Paisley, UK) and the mirVana™ Protein and RNA Isolation System (Ambion, USA), respectively, according to the manufacturers’ instructions. cDNA was synthesized using random hexamers and Moloney murine leukemia virus reverse transcriptase (Promega, Madison, USA). For BM samples, qRT-PCR was performed with the GenomeLab GeXP Genetic Analysis System (Beckman Coulter, CEQ8000, USA) using the GenomeLab™ GeXP Start Kit (Beckman Coulter, USA). The *GAPDH* gene was used as an internal control. The primer sequences were as follows: *CTCF*, 5′-AGGTGACACTATAGAATACAGCAGGAGGGTCTGCTATC-3′ and 5′-GTACGACTCACTATAGGGAGTGTGGCTTTTCATGTGACG-3′; *GAPDH*, 5′-AGGTGACACTATAGAATAAATCCCATCACCATCTTCCA-3′ and 5′-GTACGACTCACTATAGGGATTCACACCCATGACGAACAT-3′. The qRT-PCR reaction was performed with a starting temperature of 95°C for 10 min, followed by 35 cycles of 30 s at 94°C, 30 s at 55°C, and 1 min at 72°C. Each assay was repeated three times to ensure reproducibility and reliability. For cell lines, real-time PCR was done as described previously [16]. The threshold cycle (Ct) values for both *CTCF* and *GAPDH* on each PCR array were used to calculate the fold-changes in mRNA expression. The relative expression level was normalized to the *GAPDH* by the method of 2^-∆∆Ct^.

### Plasmid construction and preparation

The full-length cDNA encoding human CTCF (727 amino acids, NP_006556.1) was cloned into the pEGFP-N2 vector (EcoRI/ApaI digestion), which carries an enhanced-GFP tag; the resulting construct was named pEGFP-N2-CTCF. For the RNA interference (RNAi) experiment, the U6 promoter-driven shRNA expression vector pNeoU6 + 1 and the shRNA plasmid specific for firefly luciferase (sh-luc) were prepared by our lab facility [30]. Both plasmids contain a GFP tag. The two target sites in the *CTCF* mRNA coding regions were sh-CTCF-1 (658–677, ATGTAGATGTGTCTGTCTAC) and sh-CTCF-2 (953–971, TACTCGTCCTCACAAGTGC). These targeted sequences were verified as unique sequences in the human genomic and transcriptional sequence database (NCBI). The plasmids were purified using a Plasmid Mini Kit (Omega, Bio-tek, USA) in accordance with the manufacturer’s instructions.

### Generation of antibodies and Western blot

The anti-CTCF polyclonal antibody was generated by injecting the pET28a-CTCF antigen into a rabbit. The pET28a-CTCF antigen was constructed by inserting the N-terminal region of CTCF (amino acids 1–280) into the pET28a expression vector. The antiserum had good specificity and could be used to detect human CTCF by Western blot. The anti-GAPDH antibody was produced by the animal center of our institution [16].

Samples containing 20 μg of total protein were separated on 8% ~ 12% SDS-PAGE gels according to the different molecular weight and then transferred onto nitrocellulose membranes (Whatman, Germany) in transfer buffer (25 mM Tris-base, 40 mM glycine, and 20% methanol) using a Mini Trans-Blot Cell (BIO-RAD) at 400 mA for 2 h. The membranes were blocked by incubation in 5% nonfat milk in TBS-T (20 mM Tris, 137 mM NaCl, and 0.1% Tween 20) for 1 h at room temperature. Proteins were detected using specific rabbit polyclonal anti-CTCF (1:2,000) or rabbit polyclonal anti-GAPDH (1:5,000) antibodies. After washing with TBS-T, the membranes were incubated with goat anti-rabbit immunoglobulin G secondary antibodies (1:5,000, Pierce, USA) in TBS-T containing 5% nonfat milk for 45 min at room temperature. The proteins were visualized using an enhanced chemiluminescence kit (Amersham, USA).

### Cell culture and drug treatment

Nalm-6 is a pre-B ALL cell line with no fusion gene, while Reh is a pre-B ALL cell line with the *TEL-AML1* fusion gene. Jurkat is a T-lineage ALL cell line. Cells were cultured in a modified HyQ RPMI-1640 medium (Hyclone, USA) supplemented with 10% fetal bovine serum (FBS, PAA, USA) in a 5% CO_2_ humidified atmosphere at 37°C. In the NF-κB-inhibited drug experiments, the cells were treated with ammonium pyrrolidinedithiocarbamate (PDTC, 100 μM/ml) [31] or dimethyl sulfoxide (DMSO) for 20 h. In the NF-κB-activated drug experiments, the cells were treated with various concentrations of lipopolysaccharides (LPS, 5 or 10 μg/ml) [19] for 12 h. The cells were harvested and washed twice with PBS. The cells were incubated on ice for 30 min in 1× cell lysis buffer [20 mM Tris, 50 mM NaCl, 2 mM Na_3_VO_4_, 10 mM NaF, 1 mM EDTA, 0.1% Triton X-100, and Proteinase Inhibitor Cocktail (Roche)] and then sonicated. Following centrifugation at 4°C for 30 min, the supernatants were frozen at -80°C or used immediately.

### Nuclear protein extraction and determination of NF-κB activation

Nuclear proteins (including NF-κB p65) were isolated and analyzed by Western blot. The cells were washed twice with ice-cold PBS and suspended in NE buffer A [10 mM Hepes-NaOH (pH 7.9), 1.5 mM MgCl_2_, 10 mM KCl, proteinase inhibitor, 1 mM DTT, and 1 mM PMSF]. Intact nuclei were released from the cells by several washes with NE buffer B [NE Buffer A supplemented with 0.3% NP-40]. Nuclear membranes were damaged by adding NE buffer C [12.5% glycerol, 1mMTris-HCl (pH 6.5), 0.1 mM EDTA], followed by three cycles of sonication. A mouse monoclonal anti-p65 antibody (1:2,000, Santa Cruz, USA) was used to analyze the translocation of NF-κB to nuclei by standard Western blot analysis as described above. A rabbit polyclonal anti-histone H3 CT pan antibody (1:5,000, Upstate, USA) and a mouse monoclonal anti-α-tubulin antibody (1:10,000, Sigma, USA) were used as loading controls for nuclear and cytoplasmic proteins, respectively.

### Transient transfection, cellular apoptosis, and proliferation assays

In the knock-down and over-expression experiments, the shRNA and over-expressing plasmids were transiently transfected into Nalm-6 cells (2 × 10^6^ seeding density) using the Amaxa Cell Line Nucleofector Kit T and the Nucleofector Device (Lonza, Swiss) according to the manufacturer’s instructions. The cells were incubated for 72 h in 2 ml of antibiotic-free media containing 10% FBS and harvested for apoptosis analysis and Western blot. A total of 1 × 10^4^ cells per well were seeded in a 96-well plate after transfection, with triplicate seedings per clone. Viable cells were counted using the Cell Counting Kit-8 (CCK-8, Dojindo, Japan) assay for 5 days according to the manufacturer’s instructions. All values were normalized to the non-treated cells (sh-luc plasmid and pEFG-N2 vector). For the drug treatment experiments, PDTC or DMSO was added to the Nalm-6 cells to inhibit NF-κB activation 20 h prior to the transfection of pEGFP-N2-CTCF or pEGFP-N2. The cells were harvested for apoptosis analysis and Western blot 48 h after transfection. Cell viability was assessed by CCK-8 analysis as described above.

For the apoptotic assays, GFP-positive cells were sorted and collected by flow cytometry (BD, FACSAria II, USA) to measure the silencing efficiency. The percentages of Annexin V-APC/PI stained (BD, USA) positive and negative cells were analyzed with FlowJo software. Besides, caspase-3 activity in cells was further determined by Western blot using specific mouse monoclonal antibody against caspase-3 (Beyotime Institute of Biotechnology, Nanjing, China), which contains specificities for detecting both procaspase-3 (1:500) and cleaved caspase-3 (1:250).

### Semi-quantitative analysis

Western blots were subjected to semi-quantitative analysis using Gel-Pro Analyzer 4.0 software. The relative expression level of CTCF was normalized to the integrated optical density (IOD) of CTCF compared with GAPDH (loading control).

## Competing interests

The authors declare that they have no competing interests.

## Authors’ contributions

HZ performed cell culture, real-time PCR, cell apoptotic and proliferative assays, drug treatment experiments, pathway exploration, flow cytometry analysis and semi-quantitative analysis; LZ carried out the detection of clinical samples by qRT-PCR and Western blot, performed shRNA plasmids construction, and participated in cell apoptotic assay; Both HZ and LZ were involved in data analysis, drafted the manuscript and contributed equally in this study; HH performed over-expressing plasmids construction and anti-CTCF polyclonal antibody production; SZ carried out the bio-informatics analysis; WZ produced the heat map; XL participated in cell apoptotic assay; XZ and CG collected the clinical ALL samples and performed RNA isolation and cDNA synthesis; MM participated in the detection of clinical samples by qRT-PCR; SB conceived the idea of the study and participated in its design; HZ guided the research, participated in the study design, and revised the manuscript. All authors read and approved the final manuscript.

## References

[B1] Pui CH Mullighan CG Evans WE Relling MV Pediatric acute lymphoblastic leukemia: where are we going and how do we get there? Blood 2012 120 1165 1174 10.1182/blood-2012-05-378943 22730540 PMC3418713

[B2] Pui CH Carroll WL Meshinchi S Arceci RJ Biology, risk stratification, and therapy of pediatric acute leukemias: an update J Clin Oncol: Official J Am Soc Clin Oncol 2011 29 551 565 10.1200/JCO.2010.30.7405 PMC3071256 21220611

[B3] Downing JR Wilson RK Zhang J Mardis ER Pui CH Ding L Ley TJ Evans WE The pediatric cancer genome project Nature genetics 2012 44 619 622 10.1038/ng.2287 22641210 PMC3619412

[B4] Vostrov AA Quitschke WW The zinc finger protein CTCF binds to the APBbeta domain of the amyloid beta-protein precursor promoter. Evidence for a role in transcriptional activation J Biol Chem 1997 272 33353 33359 10.1074/jbc.272.52.33353 9407128

[B5] Filippova GN Fagerlie S Klenova EM Myers C Dehner Y Goodwin G Neiman PE Collins SJ Lobanenkov VV An exceptionally conserved transcriptional repressor, CTCF, employs different combinations of zinc fingers to bind diverged promoter sequences of avian and mammalian c-myc oncogenes Mol Cell Biol 1996 16 2802 2813 8649389 10.1128/mcb.16.6.2802 PMC231272

[B6] Bell AC West AG Felsenfeld G The protein CTCF is required for the enhancer blocking activity of vertebrate insulators Cell 1999 98 387 396 10.1016/S0092-8674(00)81967-4 10458613

[B7] Xie X Mikkelsen TS Gnirke A Lindblad-Toh K Kellis M Lander ES Systematic discovery of regulatory motifs in conserved regions of the human genome, including thousands of CTCF insulator sites Proc Natl Acad Sci U S A 2007 104 7145 7150 10.1073/pnas.0701811104 17442748 PMC1852749

[B8] Pant V Mariano P Kanduri C Mattsson A Lobanenkov V Heuchel R Ohlsson R The nucleotides responsible for the direct physical contact between the chromatin insulator protein CTCF and the H19 imprinting control region manifest parent of origin-specific long-distance insulation and methylation-free domains Genes Dev 2003 17 586 590 10.1101/gad.254903 12629040 PMC196004

[B9] Li T Hu JF Qiu X Ling J Chen H Wang S Hou A Vu TH Hoffman AR CTCF regulates allelic expression of Igf2 by orchestrating a promoter-polycomb repressive complex 2 intrachromosomal loop Mol Cell Biol 2008 28 6473 6482 10.1128/MCB.00204-08 18662993 PMC2577414

[B10] Xu N Donohoe ME Silva SS Lee JT Evidence that homologous X-chromosome pairing requires transcription and Ctcf protein Nature genetics 2007 39 1390 1396 10.1038/ng.2007.5 17952071

[B11] Tsai CL Rowntree RK Cohen DE Lee JT Higher order chromatin structure at the X-inactivation center via looping DNA Dev Biol 2008 319 416 425 10.1016/j.ydbio.2008.04.010 18501343 PMC2567126

[B12] Lobanenkov VV Nicolas RH Adler VV Paterson H Klenova EM Polotskaja AV Goodwin GH A novel sequence-specific DNA binding protein which interacts with three regularly spaced direct repeats of the CCCTC-motif in the 5′-flanking sequence of the chicken c-myc gene Oncogene 1990 5 1743 1753 2284094

[B13] Klenova EM Nicolas RH Paterson HF Carne AF Heath CM Goodwin GH Neiman PE Lobanenkov VV CTCF, a conserved nuclear factor required for optimal transcriptional activity of the chicken c-myc gene, is an 11-Zn-finger protein differentially expressed in multiple forms Mol Cell Biol 1993 13 7612 7624 8246978 10.1128/mcb.13.12.7612-7624.1993 PMC364833

[B14] Docquier F Farrar D D’Arcy V Chernukhin I Robinson AF Loukinov D Vatolin S Pack S Mackay A Harris RA Heightened expression of CTCF in breast cancer cells is associated with resistance to apoptosis Cancer research 2005 65 5112 5122 10.1158/0008-5472.CAN-03-3498 15958555

[B15] Li Z Zhang W Wu M Zhu S Gao C Sun L Zhang R Qiao N Xue H Hu Y Gene expression-based classification and regulatory networks of pediatric acute lymphoblastic leukemia Blood 2009 114 4486 4493 10.1182/blood-2009-04-218123 19755675

[B16] Zou L Zhang H Du C Liu X Zhu S Zhang W Li Z Gao C Zhao X Mei M Correlation of SRSF1 and PRMT1 expression with clinical status of pediatric acute lymphoblastic leukemia J Hematol Oncol 2012 5 42 10.1186/1756-8722-5-42 22839530 PMC3459738

[B17] Dutta J Fan Y Gupta N Fan G Gelinas C Current insights into the regulation of programmed cell death by NF-kappaB Oncogene 2006 25 6800 6816 10.1038/sj.onc.1209938 17072329

[B18] Kordes U Krappmann D Heissmeyer V Ludwig WD Scheidereit C Transcription factor NF-kappaB is constitutively activated in acute lymphoblastic leukemia cells Leukemia 2000 14 399 402 10.1038/sj.leu.2401705 10720133

[B19] Takada Y Andreeff M Aggarwal BB Indole-3-carbinol suppresses NF-kappaB and IkappaBalpha kinase activation, causing inhibition of expression of NF-kappaB-regulated antiapoptotic and metastatic gene products and enhancement of apoptosis in myeloid and leukemia cells Blood 2005 106 641 649 10.1182/blood-2004-12-4589 15811958 PMC1895177

[B20] Qi CF Martensson A Mattioli M Dalla-Favera R Lobanenkov VV Morse HC 3rd CTCF functions as a critical regulator of cell-cycle arrest and death after ligation of the B cell receptor on immature B cells Proc Natl Acad Sci U S A 2003 100 633 638 10.1073/pnas.0237127100 12524457 PMC141048

[B21] Torrano V Chernukhin I Docquier F D’Arcy V Leon J Klenova E Delgado MD CTCF regulates growth and erythroid differentiation of human myeloid leukemia cells The Journal of biological chemistry 2005 280 28152 28161 10.1074/jbc.M501481200 15941718

[B22] Rasko JE Klenova EM Leon J Filippova GN Loukinov DI Vatolin S Robinson AF Hu YJ Ulmer J Ward MD Cell growth inhibition by the multifunctional multivalent zinc-finger factor CTCF Cancer research 2001 61 6002 6007 11507042

[B23] Li T Lu L Functional role of CCCTC binding factor (CTCF) in stress-induced apoptosis Experimental Cell Res 2007 313 3057 3065 10.1016/j.yexcr.2007.05.018 PMC2706011 17583694

[B24] Fraizer G Leahy R Priyadarshini S Graham K Delacerda J Diaz M Suppression of prostate tumor cell growth in vivo by WT1, the Wilms’ tumor suppressor gene Int J Oncol 2004 24 461 471 14767530

[B25] Call KM Glaser T Ito CY Buckler AJ Pelletier J Haber DA Rose EA Kral A Yeger H Lewis WH Isolation and characterization of a zinc finger polypeptide gene at the human chromosome 11 Wilms’ tumor locus Cell 1990 60 509 520 10.1016/0092-8674(90)90601-A 2154335

[B26] Miyagi T Ahuja H Kubota T Kubonishi I Koeffler HP Miyoshi I Expression of the candidate Wilm’s tumor gene, WT1, in human leukemia cells Leukemia 1993 7 970 977 8321047

[B27] Algar EM Khromykh T Smith SI Blackburn DM Bryson GJ Smith PJ A WT1 antisense oligonucleotide inhibits proliferation and induces apoptosis in myeloid leukaemia cell lines Oncogene 1996 12 1005 1014 8649791

[B28] Courtois G Gilmore TD Mutations in the NF-kappaB signaling pathway: implications for human disease Oncogene 2006 25 6831 6843 10.1038/sj.onc.1209939 17072331

[B29] Gao J Li T Lu L Functional role of CCCTC binding factor in insulin-stimulated cell proliferation Cell proliferation 2007 40 795 808 10.1111/j.1365-2184.2007.00472.x 18021171 PMC6496666

[B30] Bao S Lu T Wang X Zheng H Wang LE Wei Q Hittelman WN Li L Disruption of the Rad9/Rad1/Hus1 (9-1-1) complex leads to checkpoint signaling and replication defects Oncogene 2004 23 5586 5593 10.1038/sj.onc.1207753 15184880

[B31] Lu L Wang L Li T Wang J NF-kappaB subtypes regulate CCCTC binding factor affecting corneal epithelial cell fate J Biol Chem 2010 285 9373 9382 10.1074/jbc.M109.094425 20110362 PMC2843186

